# Anti-Inflammatory Potential of Cultured Ginseng Roots Extract in Lipopolysaccharide-Stimulated Mouse Macrophages and Adipocytes

**DOI:** 10.3390/ijerph17134716

**Published:** 2020-06-30

**Authors:** Hyun Ju Park, Sang-Mi Jo, Seok Hee Seo, Myoungsook Lee, Yunkyoung Lee, Inhae Kang

**Affiliations:** 1 Department of Food Science and Nutrition, Jeju National University, Jeju 63243, Korea; guswnkd@naver.com (H.J.P.); angi1w@jejunu.ac.kr (S.-M.J.); bossni3@jejunu.ac.kr (S.H.S.); lyk1230@jejunu.ac.kr (Y.L.); 2 Department of Food and Nutrition, Sungshin Women’s University, Seoul 01133, Korea; mlee@sungshin.ac.kr

**Keywords:** cultured wild ginseng roots, inflammation, reactive oxygen species, antioxidant, macrophages, adipocytes, mitochondrial dysfunction

## Abstract

Wild ginseng, *Panax ginseng Meyer*, is a traditional medicine widely used in Asia. Due to low reward and high costs, wild ginseng is produced by a plant cell culture technique called cultured ginseng roots (GR). The health benefits of wild ginseng have been well studied, but the potential health effects of GR are largely unknown. Thus, we investigated the role of a GR extract (GRE) on inflammatory responses. We firstly investigated the anti-inflammatory potential of GRE in lipopolysaccharide (LPS)-stimulated RAW264.7 cells. GRE (100 μg/mL) dampened pro-inflammatory gene expression, cytokine release, reactive oxygen species (ROS) production, and mitogen-activated protein kinase (MAPK) activation. These anti-inflammatory responses by GRE were confirmed in mouse bone marrow-derived macrophages (BMDMs), which showed that GRE could inhibit inflammation with the induction of antioxidant levels. LPS was recently reported to impair mitochondrial bioenergetics in mouse macrophages. We next measured the mitochondrial oxygen consumption rate (OCR), determining mitochondrial function. LPS treatment downregulated OCR; however, GRE partially restored the LPS-mediated energy homeostasis defects. Furthermore, GRE-pretreated conditioned media (CM) obtained from mouse macrophages decreased CM-mediated adipocyte inflammation. Collectively, these data suggested that GRE attenuated LPS-induced inflammation, and it might be partially involved in the protection from mitochondrial dysfunction in macrophages and adipocytes.

## 1. Introduction

Wild ginseng (*Panax ginseng Meyer*) is well recognized as a traditional medicine in Asia, used to treat several diseases, such as neurodegenerative diseases (reviewed in [1]) and cardiovascular diseases (reviewed in [2]), as well as to improve glucose control [3], with antioxidant functions [4]. Ginseng’s beneficial health effects could be attributed to several bioactive components, such as ginsenosides, polyphenols, and flavonoids. Among them, ginsenosides, also known as ginseng saponins, are known to be the major components that have shown the most prominent health-promoting effects (reviewed in [2,5]). Ginsenosides are classified as a protopanaxadiol group (Rb1, Rb2, Rb3, Rc, Rd, Rg3, Rh2, and others), a protopanaxatriol group (Re, Rf, Rg1, Rg2, and others), or an oleanane type (Ro), depending on the aglycone skeleton [6,7]. Although wild ginseng conveys many functions that are beneficial for health, the utilization of wild ginseng is limited due to low reward and high costs. An alternative method for producing wild ginseng is by the plant cell culture of ginseng roots, known as cultured ginseng roots (GR). Several ginsenosides have been identified in GR, but the active compounds are different between wild ginseng and GR due to various environmental factors [8]. GR has been shown to inhibit inflammation [9,10] but its mechanisms of action are still unclear.

The pathogenesis of chronic disease is caused by inflammation and oxidative stress [11,12]. The cells which play an essential role in the body’s inflammatory responses are macrophages [13]. Macrophages can recognize endotoxin-signaling molecules such as lipopolysaccharide (LPS), which binds to toll-like receptor 4 (TLR4). Consequently, TLR4 activation by LPS leads to the production of a reactive oxygen species (ROS) and pro-inflammatory cytokines, including tumor necrosis factor α (TNFα), interleukin (IL)-1β, and IL-6 [14], as well as the induction of endoplasmic reticulum (ER) stress, evidenced by the activation of mitogen-activated protein kinases (MAPKs) [15]. Thus, anti-inflammatory and antioxidant drugs or components with low or no side effects, such as bioactive compounds, could be therapeutic agents for the prevention of inflammation-related diseases.

In the present study, it was hypothesized that GR extracts (GRE) could reduce inflammatory signaling by inhibiting ROS production. To address this hypothesis, two types of macrophages, murine macrophage RAW264.7 cells and bone marrow-derived macrophage (BMDM) cells, were utilized to determine GRE’s potential anti-inflammatory effects and its possible mechanism(s). Here, we are the first to report that GRE (100 μg/mL) possesses biological activities to inhibit inflammation, MAPK activation, and ROS production with antioxidant effects in mouse macrophages.

## 2. Materials and Methods 

### 2.1. Chemical Reagents

All cell cultures were purchased from SPL (Seoul, Korea). Dulbecco’s modification of Eagle’s medium (DMEM), Fetal bovine serum (FBS), and penicillin/streptomycin were purchased from Gibco (Grand Island, NY, USA). All other chemicals and reagents, including LPS (from Escherichia coli O55:B5 (L2880)), were purchased from Sigma Chemical Co. (St. Louis, MO, USA), unless otherwise stated.

### 2.2. Sample Preparation

The roots of a 4–6-year-old ginseng plant were cultivated for 55 days and then dried using a hot-air drying method (<90 °C). Dried GR was kindly provided by Joybio (Jeju, South Korea). GR was extracted using the pressurized hot water extraction method (modified from [16]), as we described previously. Briefly, a 10 g sample (dry weight) of GR was mixed with 100 mL of Milli-Q water. After this, the extracted solution was separated from the mixture by centrifugation at 3000 rpm for 3 min and then filtered using Whatman^®^ filter paper. The filtrate was lyophilized to obtain the powdered extract. Finally, the collected sample was dissolved in distilled water or dimethyl sulfoxide (DMSO, Sigma, St. Louis, MO, USA) at a concentration of 100 mg/mL with several aliquots and utilized freshly in the in vitro experiments.

### 2.3. Total Polyphenol and Flavonoid Contents of GR Extract

The total polyphenol content (TPC) of the GRE extract was analyzed using the modified Folin–Ciocalteu method [16]. The diluted sample and Folin–Ciocalteu’s phenol reagent (FMD Millipore Corporation, Darmstadt, Germany) were added into 96-well assay plates and incubated at room temperature for 5 min. Then, 4% of sodium carbonate solution (Sigma Chemical Co, St. Louis, MO, USA) was added into the plate and incubated for another 60 min at room temperature, protected from light using aluminum foil. The wavelength used in the TPC analysis was 725 nm at room temperature, and the analysis was conducted with a spectrophotometer (Molecular Devices, San Jose, CA, USA). The results were expressed as gallic acid concentration equivalents.

The analysis of the total flavonoid content (TFC) of the GRE was modified from a previously described TFC method [16]. The diluted sample and 5% sodium nitrate (Daejung Chemicals & Metals Co. Ltd., Gyeonggi-do, Korea) were added into 96-well assay plates and incubated for 6 min at room temperature. Then, 10% aluminum chloride (Daejung Chemicals & Metals Co. Ltd., Gyeonggi-do, Korea) was added and the mixture was incubated for another 5 min at room temperature. Next, 1 M of sodium hydroxide (Sigma Chemical Co, St. Louis, MO, USA) was added, and absorbance was obtained at 510 nm. The calibration curve was calculated using catechin, and the results were expressed as mg of catechin equivalent.

### 2.4. High-Performance Liquid Chromatography (HPLC) Analyses of GRE for Determining Ginsenoside Content

The International Ginseng and Herb Research Institute analyzed the content of ginsenoside (No. GHG20 190419-199#1) in GRE using HPLC (Agilent 1100 HPLC system, Santa Clara, CA, USA). The measured values (*n* = 3/ginsenoside) were calculated against the total amount of ginsenosides. The method of measurement was carried out by referring to the “Standard and Specification of Health Functional Foods” in the Notification of Professional Testing (Food and Drug Administration Notice No. 2019-10), and the method has been described previously [17].

### 2.5. Cell Culture

RAW 264.7 murine macrophage-like cells were originally obtained from the Korean Cell Line Bank (kind gift from Drs. Kim and Lee, Jeju National University, Jeju, Korea). The cells were grown in Dulbecco’s modified Eagle’s medium (DMEM), supplemented with 10% fetal bovine serum (FBS) and 1% (v/v) penicillin (100 U/mL)/streptomycin (100 μg/mL) under a humidified condition of 5% CO_2_ at 37 °C. RAW264.7 cells were pretreated with GRE for 48 h, then starved in DMEM for 12–18 h before stimulation of LPS (1 μg/mL) for 2 h in DMEM in the presence or absence of GRE. An inflammatory response was induced by LPS (purchased from Sigma-Aldrich Chemical Co.).

The primary bone marrow was isolated from C57BL/6 mice. The method was modified from [18]. Briefly, bone marrow was obtained from the femur of the mouse, and the cells were suspended in DMEM containing 20% FBS, penicillin (100 U/mL), and streptomycin (100 μg/mL), in the presence of 30% L929cell-conditioned medium. The same media were used for the culture of the BMDMs. Differentiated macrophages (BMDM) were allowed to attach and form a monolayer for 7–10 days. All protocols and procedures were approved by the Institutional Animal Care and Use Committee at Jeju National University (Approval ID # 2019-0004).

The 3T3-L1 cells (American Type Culture Collection (ATCC), VA, USA) were grown as described previously [16]. Briefly, to make confluence in a basal medium, DMEM with penicillin/streptomycin and 2 mM L-glutamine (Sigma) was supplemented with 10% newborn calf serum (Linus, Madrid, Spain). Two days after the cells reached confluence (referred to as Day 0), they were induced to differentiate in a basal medium containing 10% Gibco (Grand Island, NY, USA), 1 μM dexamethasone (Sigma), 0.5 mM 3-isobutyl-1-methylxanthine (Sigma), and 2 nM insulin (Sigma) for 48 h. This was followed by 48 h in a basal medium containing 10% FBS and 2 nM insulin. The cells were subsequently refed fresh basal medium, supplemented with 10% FBS (without insulin), every other day. 

### 2.6. Total RNA Extraction and qPCR

Gene-specific primers for qPCR were obtained from Cosmo Genetech (Korea). Total RNA was isolated with Trizol reagent (Invitrogen, Carsbad, CA, USA). To remove potential genomic DNA contamination, mRNA was treated with DNase (Mediatech). RNA concentrations were measured by the NanoDrop (Nano-200 Micro-Spectrophotometer, Hangzhou City, China). A total of 1 μg of mRNA was converted into cDNA in a total volume of 20 μl (high-capacity cDNA reverse transcription kits, Applied Biosystem, USA). Gene expression was determined by real-time qPCR (CFX96™ Real-Time PCR Detection System, Bio-Rad, USA) and relative gene expression was normalized by hypoxanthine-guanine phosphoribosyltransferase (HPRT) and/or ribosomal protein lateral stalk subunit P0 (RPLP0, 36B4) (primer sequences are shown in Table 1).

### 2.7. Western Blot Analysis

RAW264.7, BMDM, and 3T3-L1 adipocytes were scraped with an ice-cold radioimmune precipitation assay (RIPA) buffer (Thermo Scientific, Waltham, MA, USA) containing protease and phosphatase inhibitors (Sigma) in order to prepare the total cell lysates. Proteins were separated using 8% or 10% sodium dodecyl sulfate-polyacrylamide gel electrophoresis (SDS-PAGE), transferred to polyvinylidene difluoride (PVDF) membranes, and incubated with the relevant antibodies. Chemiluminescence from the enhanced chemiluminescence (ECL, Western Lightning) solution was detected with the ChemiDoc (Bio-Rad, Hercules, CA, USA) system. Polyclonal or monoclonal antibodies targeting phospho-JNK (#4668), ERK (#4370), p38 (#4511), total ERK (#4695), NF-kB (#8242), and β-actin (#4967) were purchased from Cell Signaling Technology (Beverly, MA, USA).

### 2.8. Inflammation Cytokine Detection by ELISA

RAW264.7 and BMDM were pretreated with GRE for 48 h. Later, the cells were stimulated with lipopolysaccharide (LPS, Sigma, St. Louis, MO, USA) at a concentration of 1 μg/mL or 100 ng/mL for 2–4 h, respectively. Collected media were centrifuged and measured for inflammatory cytokine. TNF-α (BD PharMingen, San Jose, CA, USA) ELISA kits were used according to their protocols. Absorbance was measured at 450–570 nm by an ELISA microplate reader (Spectra Max i3X, Molecular Devices, San Jose, CA, USA).

### 2.9. Reactive Oxygen Species (ROS) Detection

Intracellular ROS was measured by the 2,7-dichloro-dihydro-fluorescein diacetate (DCFDA) cellular ROS detection assay kit (Abcam, Cambridge, UK) in RAW264.7 cells. Briefly, RAW264.7 cells were pre-incubated with either DMSO or GRE (100 μg/mL) for 48 h, and then the RAW264.7 cells were treated with LPS (1 μg/mL), with or without GRE. After 2–4 h, RAW264.7 cells were washed with Hanks’ balanced salt solution (HBSS) and loaded with 20 μM DCFDA for 1 h at 37 °C. After 1 h incubation, HBSS was used to wash out the unincorporated dye. Then, 250 μM of H_2_O_2_ (t-butyl hydroperoxide (Sigma Aldrich, St. Louis, MO, USA)) was spiked into the RAW264.7 cells with experimental conditioned media in the presence or absence of GRE. An ELISA microplate reader measured fluorescence intensity. The oxidation of DCFDA to the highly fluorescent 2,7-dichlorofluorescein (DCF) was proportionate to ROS generation.

### 2.10. Oxygen Consumption Rate (OCR) by Seahorse

Mitochondrial respiration activities detected by the O_2_ consumption were measured using an XF24 extracellular flux analyzer (Agilent Technologies, Santa Clara, California, USA), as described previously [16], in the RAW264.7 cells. Briefly, the RAW264.7 cells were seeded in a Seahorse microplate (24-well) and preincubated with GRE (100 μg/mL) or DMSO for 48 h, and LPS (1 μg/mL) was loaded for 2–4 h. The cells were then treated with oligomycin (oligo, 2 μM) to measure the ATP turnover. The maximum respiratory capacity was assessed by the addition of carbonyl cyanide 4-trifluoromethoxy phenylhydrazone (FCCP, 0.5 μM), which is a chemical uncoupler of electron transport and oxidative phosphorylation [19]. The mitochondrial respiration was blocked by a combination of antimycin A (1 μM) and rotenone (1 μM) (ROT/AA). All chemicals were purchased from Agilent (Seahorse XF cell mito stress test kit, 103015-100, Santa Clara, CA, USA). The calculated OCR value was normalized by the protein concentration and expressed as pmol O_2_/minutes/μg protein.

### 2.11. Statistical Analysis

All the data were expressed as the means ± standard error of mean (SEM), and statistical calculations were performed using the t-test and ANOVA (one-way analysis of variance) with Bonferroni’s multiple comparison tests. Results were considered significant if *p* < 0.05 (Graph Pad Prism Version 7.0, La Jolla, CA, USA).

## 3. Results

### 3.1. Total Polyphenol, Flavonoid, and Ginsenoside Contents of GR Extract

We firstly prepared GR powder and then extracted GR by the pressurized hot water extraction method (10g sample in 100 mL of Milli-Q water). The GR solution was filtered, lyophilized, powdered, and then dissolved in distilled water or dimethyl sulfoxide (DMSO) (GRE, stock 100 mg/mL). Extracted GR was analyzed for the total polyphenol content and total flavonoid content. The total polyphenol content of GRE was equivalent to 10.87 mg gallic acid/g extract, while the total flavonoid content of GRE was equivalent to 3.79 mg catechin/g extract (Table 1, Upper). We also measured the ginsenoside content by high-performance liquid chromatography (HPLC). The ginsenoside (Rg1, Re, Rh1(S), Rg2(S), Rg2(R), Rb1, Rc, Rb2, Rg6, Rg3(S), Rg3(R), Rh2(S)) content of GRE is shown in Table 1 (Table 2, down). Re, Rb1, and Rg1 were among the most abundant ginsenosides in GRE.

### 3.2. GR Extract Attenuated LPS-Induced Inflammatory Responses, ER Stress, and Reactive Oxygen Species in Mouse Macrophage Cell Line, RAW264.7 Cells

**Figure 1:**
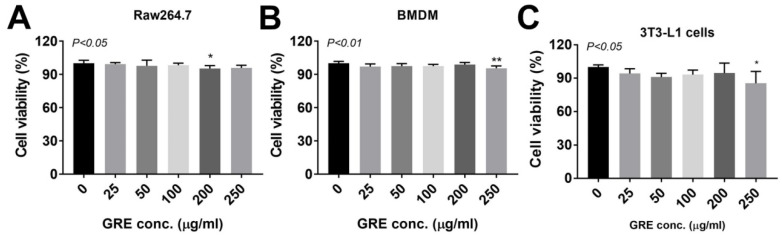
Effects of cultured ginseng roots (GR) extract on cell viability in RAW264.7, mouse bone-marrow-derived macrophages (BMDM), and 3T3-L1 pre-adipocytes. The culture of (**A**) RAW264.7, (**B**) BMDM, and (**C**) 3T3-L1 were treated with 25–250 μg/mL of GRE for 24 h. 2,3-Bis-(2-Methoxy-4-Nitro-5-Sulfophenyl)-2H-Tetrazolium-5-Carboxanilide (XTT) reagent was added 3 h before measurement of OD 450nm. Data are expressed as percentages of the vehicle control (dimethyl sulfoxide (DMSO)). Data are represented as the mean ± SEM of three independent experiments (*n* = 8/group for each experiment); * *p* < 0.05; ** *p* < 0.01; compared with vehicle vs. GRE-treated group by one-way ANOVA with Bonferroni’s comparison test.

Next, we checked the cell viability to identify the cytotoxic effects of GRE (25–250 μg/mL) in a different cell type (RAW264.7, BMDM, and 3T3-L1 cells). There was no significant reduction of up to 100 μg/mL of GRE in any of the three different cells. However, 200 μg/mL and 250 μg/mL of treatment were shown to have cytotoxic events in RAW264.7 cells and BMDM/3T3-L1 cells, respectively (Figure 1) (RAW264.7 cells 0 vs. 200 μg/mL GRE: 100 ± 0.96 vs. 95.36 ± 0.90, *p* < 0.05/ BMDM 0 vs. 250 μg/mL GRE: 100 ± 0.63 vs. 95.58±0.72, *p* < 0.01/ 3T3-L1 cells 0 vs. 200 μg/mL GRE: 100 ± 1.13 vs. 85.31 ± 3.789, *p* < 0.05). Next, we set up the inflamed macrophage and adipocyte models. Inflammation was stimulated by using 1 μg/mL concentration of LPS in RAW264.7 cells [20,21] and 100 ng/mL of LPS for BMDM [22] and 3T3-L1 adipocytes [23], which are well-accepted concentrations in each type of cell. 

**Figure 2:**
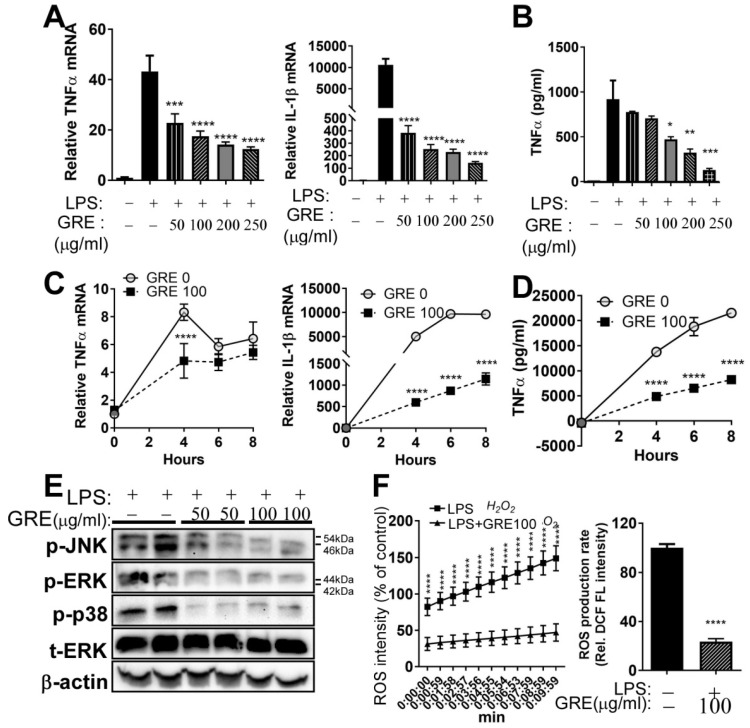
GR extract attenuated lipopolysaccharide (LPS)-induced inflammatory responses, endoplasmic reticulum (ER) stress, and reactive oxygen species (ROS) in mouse RAW264.7 macrophage cell lines. RAW264.7 cells were pretreated with GRE (50–250 μg/mL) for 48 h in FBS-containing completed media and then starved for 12–18 h in DMEM, followed by LPS stimulation (1 μg/mL) for 2 h in the presence or absence of GRE. (**A**) TNFα and IL-1β gene expression by qPCR. (**B**) TNFα cytokine production by ELISA; cells were pretreated with GRE (100 μg/mL) for 48 h, followed by LPS stimulation (1 μg/mL) for 4, 6, and 8 h. (**C**) TNFα and IL-1β gene expression by qPCR. (**D**) TNFα cytokine production by ELISA. (**E**) Protein extracts from LPS-treated RAW264.7 cells were immunoblotted with antibodies targeting phosphor (p)-JNK, p-ERK, p-p38, total (t)-ERK, or β-actin (loading control). (**F**) Relative ROS production in RAW264.7 with or without GRE, using 2′,7′–dichlorofluorescein (DCF) fluorescence for detecting ROS production. All values are presented as the mean ± SEM (*n* = 3–4/group for each experiment); * *p* < 0.05; ** *p* < 0.01; *** *p* < 0.001; **** *p* < 0.0001; compared with LPS-treated group vs. LPS + GRE-treated group by one-way ANOVA with Bonferroni’s comparison test. +, treatment; −, non-treatment.

Emerging evidence has shown that GR has anti-inflammatory properties in mouse macrophage RAW264.7 cells [9,10]. In our study, LPS stimulation significantly increased mRNA levels of TNFα and IL-1β, which were significantly decreased by GRE treatment (50–250 μg/mL) in RAW 264.7 macrophages (Figure 2A). To further determine the production of pro-inflammatory cytokine by GRE, TNFα ELISA was used in the culture supernatants. As seen in Figure 2B, the levels of TNFα secretion were impeded dose-dependently with GRE treatment, compared to the LPS control. Furthermore, TNFα and IL-1β expression were increased in a time-dependent manner, while GRE significantly decreased (Figure 2C). Consistently, the highest TNFα cytokine release was at 8 h after LPS stimulation, after which the levels were inhibited by GRE (Figure 2D).

It is well known that ER stress regulates pro-inflammatory cytokines by altering MAPK signaling [24,25]. To investigate the effects of GRE on the LPS-induced activation of MAPKs, such as extracellular signal-regulated kinase (ERK), c-jun NH2-terminal kinase (JNK), and p38 MAPK, we measured protein expression by Western blot analysis. GRE attenuated the phosphorylation of ERK, JNK, and p38 in a concentration-dependent manner (Figure 2E).

Oxidative stress inhibition is closely associated with anti-inflammatory responses [26]. We then speculated that GRE could reduce LPS-mediated oxidative stress in mouse macrophage cells. To address this, we determined the ROS production by measuring 2′,7′–dichlorofluorescein (DCF) fluorescence levels in the presence or absence of GRE (100 μg/mL). Increased ROS production was completely dampened by GRE pre-treatment in RAW 264.7 cells, which indicated that GRE prevented LPS-induced oxidative stress (Figure 2F).

### 3.3. GR Extract Attenuated LPS-Induced Inflammatory Responses and ER Stress while Increasing Antioxidant Gene Expression in Mouse BMDMs

**Figure 3:**
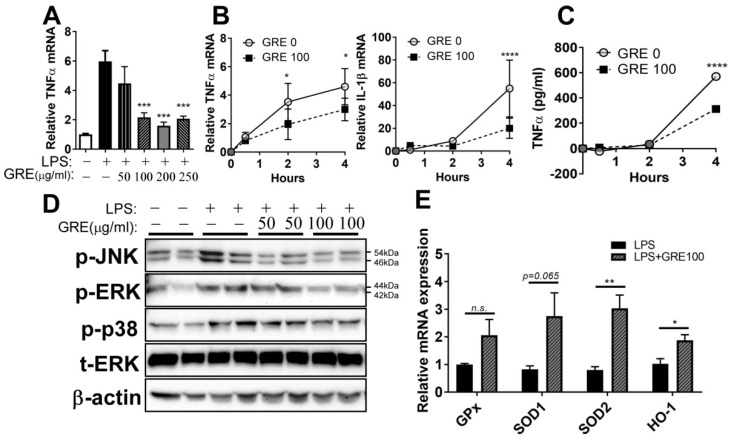
GR extract attenuated LPS-induced inflammatory responses and ER stress with the induction of antioxidant gene expression in mouse bone-marrow-derived macrophages (BMDMs). BMDMs were pretreated with GRE (50–250 μg/mL) for 48 h in FBS-containing completed media and then starved in DMEM for 12–18 h, followed by LPS stimulation (100 ng/mL) for 2 h (A,D) or 0.5, 2, and 4 h (B,C), in the presence or absence of GRE. (**A**) TNFα gene expression by qPCR. (**B**) TNFα and IL-1β gene expression by qPCR. (**C**) TNFα cytokine production by ELISA. (**D**) Protein extracts from LPS-treated BMDMs were immunoblotted with antibodies targeting p-JNK, p-ERK, p-p38, t-ERK, or β-actin (loading control); BMDMs were pretreated with GRE (100 μg/mL) for 48 h in FBS-containing completed media and then starved in DMEM, followed by LPS stimulation (100 ng/mL) for 4 h in the presence or absence of GRE. (**E**) GPx, SOD1, SOD2, and HO-1 gene expression by qPCR. All values are presented as the mean ± SEM (*n* = 3–4/group for each experiment). * *p* < 0.05; ** *p* < 0.01; *** *p* < 0.001; **** *p* < 0.0001; compared with LPS-treated group vs. LPS + GRE-treated group by one-way ANOVA with Bonferroni’s comparison test. +, treatment; −, non-treatment.

The anti-inflammatory properties of GRE in RAW264.7 cells were confirmed by using primary bone-marrow-derived macrophage (BMDM) cells from mice. A 100 ng/mL volume of LPS was used for stimulating inflammation, based on other literature [22]. Pre-treatment GRE repressed the gene expression of TNFα in LPS-stimulated cells (Figure 3A). In the time-dependent experiments, TNFα and IL-1β expression were increased in a time-dependent manner, which reached the maximal levels at 4 h by LPS, while GRE significantly decreased (Figure 3B). In a consistent manner, TNFα cytokine release was the highest 4 h after LPS stimulation, after which the levels were drastically decreased by GRE (Figure 3C).

We next wondered whether GRE downregulated LPS-mediated ER stress by inhibiting MAPK activation. Phosphorylation of ERK, JNK, and p38 MAPK was increased by LPS stimulation compared with the control group. GRE pre-treatment was effective in attenuating the phosphorylation of MAPKs, especially at a volume of 100 μg/mL in the GRE treated group (Figure 3D).

Since GRE inhibited the production of ROS (Figure 2F), it was assumed that pre-treatment with GRE could promote antioxidant enzyme expression in macrophages. The expression of antioxidant genes, such as glutathione peroxidase (GPx), superoxide dismutase 1 (SOD1), SOD2, and heme oxygenase 1 (HO-1), was examined. BMDM showed an increase in the expression of SOD2 and HO-1 genes following treatment with GRE, compared to LPS treatment alone (Figure 3E). The levels of GPx and SOD1 were also marginally increased by GRE but were not significantly different. These results indicated that the prevention of ROS and upregulation of antioxidant enzymes are closely linked to the anti-inflammatory properties of GRE in mouse macrophages.

### 3.4. GR Extract Protected Mitochondrial Function and Energy Metabolism in LPS-Induced Inflamed Macrophages

**Figure 4:**
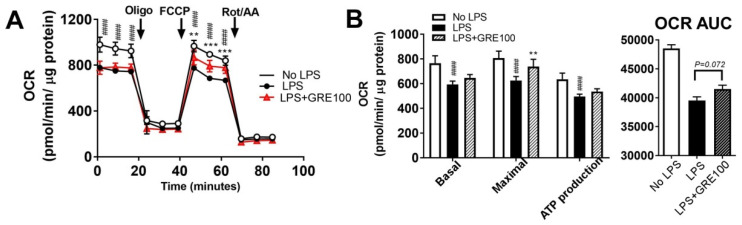
GR extract protected from LPS-mediated impairment of energy metabolism. RAW264.7 cells were pretreated with GRE (100 μg/mL) for 48 h in FBS-containing completed media and then starved in DMEM for 12–18 h, followed by LPS stimulation (1 μg/mL) for 2–4 h in the presence or absence of GRE. (**A**,**B**) Oxygen consumption rate (OCR) in RAW264.7 cells treated with vehicle without LPS (white circle, white bar), vehicle with LPS (black circle, black bar), and LPS + GRE (grey triangle, grey bar), as determined by Seahorse extracellular analyzer. Arrow indicates the treatment of inhibitors of oligomycin (Oligo), carbonyl cyanide 4-trifluoromethoxy phenylhydrazone (FCCP), and a combination of antimycin A and rotenone (Rot/AA). All values are presented as the mean ± SEM (*n* = 5/group for each experiment). ^####^ *p* < 0.0001; compared with non-LPS-treated group vs. LPS-treated group. ** *p* < 0.01; *** *p* < 0.001; compared with LPS-treated group vs. LPS + GRE-treated group by one-way ANOVA or two-way ANOVA with Bonferroni’s comparison test.

Maintaining energy homeostasis in immune responses is requisite in macrophages [27,28]. To gain an insight into whether reduced inflammation by GRE helps to maintain the energy metabolism in macrophages, the mitochondrial oxygen consumption rate was measured by the Seahorse extracellular analyzer. The LPS-stimulated inflammation impaired the basal/maximal respiration rate and mitochondrial adenosine triphosphate (ATP) production compared to the non-LPS-stimulated group. GRE was able to maintain mitochondrial function by enhancing maximal cellular respiration, compared to the LPS-treated group (Figure 4A,B). These results indicated that the anti-inflammatory potential of GRE might contribute to the protection of mitochondrial function.

### 3.5. GR Extract Attenuated LPS-Mediated Adipocyte Inflammation

**Figure 5:**
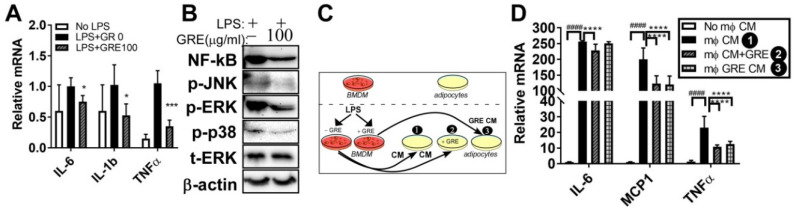
GR extracts attenuated the LPS-induced inflammatory responses in 3T3-L1 adipocytes and adipocytes exposed to macrophage-conditioned media (CM). The 3T3-L1 cells were seeded and induced to differentiation in the presence of dimethyl sulfoxide (DMSO, vehicle control) and GRE (100 μg/mL) for 7 days and then starved in DMEM for 12–18 h, followed by LPS stimulation (100 ng/mL, 2h) in the presence or absence of GRE. (**A**) IL-6, IL-1β, and TNFα gene expression by qPCR. (**B**) Protein extracts from LPS-treated 3T3-L1 adipocytes in the presence or absence of GRE (100 μg/mL) were immunoblotted with antibodies targeting NF-kB, p-JNK, p-ERK, p-p38, t-ERK, or β-actin (loading control); 3T3-L1 adipocytes were preincubated with DMSO or GRE before receiving the CM of LPS-treated BMDMs with or without GRE. (**C**) Experimental scheme. (**D**) IL-6, MCP1, and TNFα gene expression by qPCR. All values are presented as the mean ± SEM (*n* = 3–4/group for each experiment). ^####^ *p* < 0.0001; compared with non-macrophage (mφ) CM vs. mφ CM. * *p* < 0.05; *** *p* < 0.001; **** *p* < 0.0001 compared with LPS-treated group or m mφ CM vs. GRE-treated group by one-way ANOVA with Bonferroni’s comparison test. +, treatment; −, non-treatment.

Obesity and low-grade chronic inflammation of adipocyte are closely linked by increasing macrophage infiltration and inflammatory response [29]. To investigate whether GRE inhibits adipose inflammation, LPS (100 ng/mL) was stimulated in the fully differentiated adipocytes, with or without GR extracts (100 μg/mL). The upregulated mRNA levels of pro-informatory genes IL-6, IL-1β, and TNFα by LPS was attenuated by GRE treatment (Figure 5A). Next, we examined whether GRE treatment reduces nuclear factor kappaB (NF-kB), which is a major transcription factor of inflammation and MAPK activation and plays a pivotal role in adipocyte inflammation [30]. The upregulation of NF-kB and the phosphorylation levels of the three MAPKs (i.e., p-JNK, p-p38, and p-ERK) by LPS were blocked by GRE (Figure 5B).

To further examine the cross-talk between murine macrophages and adipocytes, we examined whether the GRE treatment of macrophages would prevent macrophage conditioned medium (CM)-mediated adipocyte inflammation. Therefore, 3T3-L1 cells were cultured with or without GRE during adipogenesis and then the adipocyte cultures were exposed to the macrophage-CM. CM was obtained after incubation of BMDM with LPS (100 ng/mL, 4h), alone or CM-obtained after GRE pre-treatment (100 μg/mL, 48h) (Group 1: adipocytes exposed to CM; Group 2: adipocytes cultured with GRE and exposed to CM; Group 3: adipocytes exposed to GRE-pretreated CM) (Figure 5C). The expression of inflammatory genes (IL-6, MCP1, and TNFα) was drastically increased following exposure to the macrophage-CM. Adipocytes which had received GRE reduced IL-6, MCP1, and TNFα gene expression following exposure to the CM (Group 1 vs. Group 2). Adipocytes exposed to GRE-pretreated CM reduced MCP1 and TNFα levels when compared with CM-exposed adipocytes (Group 1 vs. Group 3) (Figure 5D). These data indicate that the inhibition of MAPK/NF-kB activation by GRE is involved in the attenuation of inflammation in 3T3-L1 adipocytes.

## 4. Discussion

Host defensive systems could be disrupted by the aberrant production of inflammatory signaling, pro-inflammatory gene expression, ROS, and cytokine production in macrophages [12,31]. LPS, the major component of the outer membrane of Gram-negative bacteria [32], binds to toll-like receptor 4 (TLR4), which leads it to recruit NF-kB [33]. The activation of the NF-kB-dependent pathway promotes inflammation-associated gene activity, resulting in the activation of the MAPK pathways [12]. Identifying the therapeutic agents has protective effects against inflammatory diseases; however, risk factors are necessary. In this study, we addressed two critical questions: 1) does GRE attenuate LPS-mediated inflammation in macrophages and what mechanisms are involved, and 2) does GRE also inhibit adipocyte inflammation? To address these questions, we performed in vitro studies using mouse macrophages, namely RAW264.7 cells and primary BMDM and 3T3-L1 adipocytes, and we found that GRE mitigates the pathophysiological consequences of inflammation.

Regarding the first question, we found that GRE significantly reduced inflammatory responses and MAPK activation both in LPS-treated RAW264.7 cells (Figure 2) and BMDMs (Figure 3). These results are consistent with a report by Park et al. They extracted two types of cultured ginseng roots (non-fermented vs. fermented) and showed that fermented, cultured ginseng roots possessed high anti-inflammatory activity in RAW264.7 cells, especially at a dose of 2.5 mg/mL [9]. In addition to in vitro systems, the oral administration of GR to guinea pigs also effectively inhibited ROS concentration and lipid peroxidation and increased antioxidant capacity [34]. i) The first mechanisms that we think to be involved in the anti-inflammatory effects of GRE in macrophages are antioxidant properties. In our study, LPS-stimulated ROS production was blocked by GRE (Figure 2F), which might be involved in the antioxidant properties of GRE (Figure 3E). Consistently, Yu et al. also reported that 100 μg/mL of GRE concentration effectively reduced inflammatory expression with the activation of nuclear factor (erythroid-derived 2)-like 2 / HO-1 expression and the inhibition of nitric oxide (NO) production in inflamed RAW264.7 cells by LPS [10]. Antioxidants neutralize oxidative stress, which leads to a decrease in excess free radicals, and they protect cells by reducing inflammation [35]. There are many plant extracts which have shown antioxidant and anti-cancer activities by inhibiting free radicals and lipid peroxidation [36]. However, the antioxidant/anti-inflammatory mechanisms of action of GRE are still unclear. Thus, we are currently performing a gain of function and loss of function strategy in order to identify the pathways involved in the anti-inflammatory action of GRE. ii) The second mechanism that we think to be involved in the anti-inflammatory effects of GRE in macrophages is the modulation of mitochondrial function. Recent findings support the notion that mitochondrial respiratory functions and energy production are repressed by ROS [37]. Moreover, LPS is well documented as dampening mitochondrial function, which is associated with an inflammatory phenotype (M1) [38]. Our results showed that GRE attenuated energy homeostasis by preventing the downregulation of mitochondrial oxygen consumption (Figure 4). It suggested that the upregulation of mitochondrial function may be involved in the protection from LPS-mediated inflammation in macrophages. Consistent with our results, Kim et al. showed that gamma tocotrienol treatment sustains the ATP production capacity (oxygen consumption and glycolysis capacity) against LPS-induced inflammation [18], which indicated that functional nutrients and/or food might be able to restore energy homeostasis that has been impaired by LPS. The cell model in our study that was used to check the energy capacity was RAW264.7 cells, a cell line which does not represent real macrophage metabolism; thus, further investigation is warranted by using BMDM or primary human macrophages in order to confirm mitochondrial function. Our group is also planning to conduct a separate experiment to identify whether GRE favors monocyte polarization into M2 macrophages.

Regarding the second question, we have demonstrated that GRE reduced LPS-induced NF-kB levels, MAPK activation, and inflammatory markers (IL-6, IL-1β, and TNFα) in adipocytes (Figure 5A,B). Moreover, GRE revealed an anti-inflammatory role in adipocytes, which were incubated in a macrophage-conditioned medium (Figure 5C,D). Adipose inflammation occurred with adipose tissue remodeling, collagen deposition, and macrophage infiltration [39]. Numerous studies demonstrated that ginseng and/or ginsenosides suppressed obesity and adipocyte inflammation by regulating several pathways, such as matrix metalloproteinases (MMP)-2 and MMP-9 [40], AMPK signaling [41], and/or PPAR-γ signal pathways [42,43]. However, to date, no studies have investigated the effects of GRE on adipocyte inflammation. It has been reported that cultured wild ginseng roots contain high amounts of ginsenoside, which are compatible with the amounts found in wild ginseng, but in a different ginsenoside ratio [6]. This notion led us to assume that the anti-inflammatory effects of GRE both in adipocytes and macrophages were maybe due to the action of its active components, ginsenosides. Although the ginsenoside content of GRE was measured, the amount of ginsenosides was small. Further study is warranted in order to identify, particularly, the other bioactive components, besides ginsenosides, that are present in GR extracts.

The major limitations of our study are (i) the similarity of the research question to several other studies (Park et al. [9], Yu et al. [10], and our study), namely, whether GR could attenuate inflammatory responses. However, we firstly identified that GRE impeded inflammation by reducing inflammatory gene/cytokine release and ER stress in mouse macrophages and even in adipocytes. We also identified that GRE’s antioxidant and mitochondrial maintenance capacities were major contributors to the anti-inflammatory properties of GRE. Another limitation of our research is that (ii) it is still unclear what specific compartments are most responsible for the anti-inflammatory function of GRE. We are currently adapting the separation methods of phenolic compounds of GRE to unravel these issues. (iii) Lastly, it is uncertain whether 100 µg/mL of GRE is a clinically relevant concentration. Although the concentration in our study (100 µg/mL) was smaller than other studies (up to 2.5 mg/mL) and showed no cytotoxic effects in various cell types (Figure 1), investigating the clinical adequacy and efficacy of GRE supplementation is required in order to unravel these issues. Nevertheless, to our knowledge, this is the first study to show that GRE suppresses the aberrant production of pro-inflammatory signaling induced by LPS, including gene expression, cytokine release, ROS production with antioxidant properties, and protection of the mitochondrial dysfunction against LPS-driven inflammation.

## 5. Conclusions

GRE attenuated LPS-induced inflammation, including gene expression, cytokine release, and ROS production with antioxidant properties, and it might be partially involved in the protection of mitochondrial dysfunction in macrophages and adipocytes.

## Figures and Tables

**Table 1:** Primer sequences for qPCR.

Gene	Forward/Reverse	Sequence (5′–3′)
mGPx	Forward	AGTCCACCGTGTATGCCTTCT
	Reverse	GAGACGCGACATTCTCAATGA
mHO-1	Forward	CAGGTGATGCTGACAGAGGA
	Reverse	TCTCTGCAGGGGCAGTATCT
mHPRT	Forward	TTGCTCGAGATGTCATGAAGGA
	Reverse	AGCAGGTCAGCAAAGAACTTATAGC
mIL-1β	Forward	AAATACCTGTGGCCTTGGGC
	Reverse	CTTGGGATCCACACTCTCCAG
mIL-6	Forward	CTGCAAGAGACTTCCATCCAGTT
	Reverse	AGGGAAGGCCGTGGTTGT
mMCP1	Forward	AGGTCCCTGTCATGCTTCTG
	Reverse	GCTGCTGGTGATCCTCTTGT
mSOD1	Forward	AACCAGTTGTGTTGTCAGGAC
	Reverse	CCACCATGTTTCTTAGAGTGAGG
mSOD2	Forward	CAGACCTGCCTTACGACTATGG
	Reverse	CTCGGTGGCGTTGAGATTGTT
mTNFα	Forward	GGCTGCCCCGACTACGT
	Reverse	ACTTTCTCCTGGTATGAGATAGCAAAT
m36B4	Forward	GGATCTGCTGCATCTGCTTG
	Reverse	GGCGACCTGGAAGTCCAACT

**Table 2:** Total polyphenol, flavonoid, and ginsenoside contents of ginseng roots extract (GRE).

	GRE (mg/mL)
Total polyphenols (mg gallic acid/extract g)	10.87 ± 0.11
Total flavonoids (mg catechin/extract g)	3.79 ± 0.14 ^1^
Rg1	0.11 ± 0.00
Re	0.27 ± 0.01
Rh1 (S)	0.02 ± 0.00
Rg2 (S)	0.08 ± 0.00
Rg2 (R)	0.06 ± 0.00
Rb1	0.16 ± 0.01
Rc	0.05 ± 0.00
Rb2	0.04 ± 0.00
Rg6	0.01 ± 0.00
Rg3 (S)	0.01 ± 0.00
Rg3 (R)	0.03 ± 0.001
Rhs (S)	Non-detected

^1^ Values are presented as the mean ± SEM of three independent experiments.
